# Clinical and radiographic presentation of pelvic sarcoma in children

**DOI:** 10.1051/sicotj/2018040

**Published:** 2018-10-19

**Authors:** Muayad Kadhim, Nariman Abol Oyoun, Richard B. Womer, John P. Dormans

**Affiliations:** 1 Department of Orthopaedic Surgery, Louisiana State University Health Science Center, Children's Hospital of New Orleans, New Orleans, LA 70112 USA; 2 Department of Orthopeadic Surgery, Assiut University Hospital, Assiut Egypt; 3 Department of Pediatrics, Children's Hospital of Philadelphia, Philadelphia, PA 19104 USA; 4 Department of Orthopaedics, Indiana University School of Medicine, Indianapolis, IN 46202 USA

**Keywords:** Pelvic sarcoma, Ewing Sarcoma, Osteosarcoma, Undifferentiated sarcoma, Rhabdomyosarcoma, Synovial cell sarcoma, Fibrosarcoma, Dermatofibrosarcoma, Fibromyxoid sarcoma, Chondrosarcoma, Chordoma and epithelioid sarcoma, Lytic lesion, Sclerotic lesion

## Abstract

*Introduction*: Pelvic sarcomas may present with vague symptoms. The aim of this study was to examine the characteristics and clinical presentations of different types of pelvic sarcoma in children.

*Methods*: This is a retrospective cohort study of patients up to 21 years of age with the diagnosis of pelvic sarcoma between January 2000 and June 2013. Data on demographics, tumor type and location, and clinical presentation were collected from the medical records.

*Results*: A total of 59 patients [37 males (62.7%) and 22 females (37.3%)] were examined in this study. Mean age at presentation was 11.3 ± 5 years (range 0.8–21 years). Thirty-six patients had Ewing sarcoma (61%), 9 osteosarcoma (6.8%), 4 undifferentiated sarcoma (6.8%), 2 (3.4%) rhabdomyosarcoma, 2 synovial cell sarcoma, and one (1.7%) of each fibrosarcoma, dermatofibrosarcoma, fibromyxoid sarcoma, chondrosarcoma, chordoma, and epithelioid sarcoma. Pain at presentation was reported in 41 patients, 13 mass, 8 limping, and 5 neurologic symptoms. Most of the bony tumors were painful (77%), while most of the soft tissue tumors were painless (70%). Nine patients presented with constitutional symptoms. Most patients presented within 4–12 months from symptoms beginning. Twenty-one patients (35.6%) presented with metastases (14 Ewing sarcoma, 6 osteosarcoma, and 1 synovial cell sarcoma). Pelvic radiographs showed lytic lesion in 11 patients, 4 sclerotic lesions, 6 mixed lesion, 6 had only soft tissue mass, 1 radiograph showed osteopenia, and 2 radiographs were reported normal.

*Conclusion*: Ewing sarcoma was the most common pelvic sarcoma tumor in children. In most cases, pelvic sarcoma in children presented with pain mimicking other benign conditions. Some patients presented with metastatic disease with no prognostic clinical or radiographical signs or symptoms. Pelvic sarcoma should be considered a differential diagnosis as part of children work up.

## Introduction

Malignant bone and soft tissue tumors are rare in children [1–4]. Osteosarcoma and Ewing sarcoma are the most common bone sarcoma [4,5], while Rhabdomyosarcoma is the most common soft tissue sarcoma constituting more than 50% of soft tissue sarcomas [6]. The peak of incidence varies between these sarcoma types. The first peak of osteosarcoma is in children and adolescents and the second peak is in elderly patients related to Paget disease [4,7,8]. Most of Ewing sarcoma cases occur in the second decade (85%) [7]. More than 60% of Rhabdomyosarcoma in children happens in the first decade with slight increase in incidence in late adolescent 20% [6]. Anatomic location preference is also different between these tumors. Osteosarcoma is mostly located in the long bones [4,7]. Only 4%–13% of osteosarcoma in children occurs in the pelvis [4,5,7–10]. On the other hand, 33% of Ewing sarcoma occur in the lower extremities, 24% in the pelvis, and 12% in the thorax [11]. Laitinen et al. examined pelvic sarcoma in children and reported that within the pelvic bone, Ewing sarcoma rate was 78% compared to Osteosarcoma 22% [5].

Children with pelvic sarcomas may present with vague symptoms because of the complex anatomy and proximity to multiple systems, which may delay the diagnosis. The aim of this study was to review all cases of musculoskeletal bone and soft tissue sarcoma of the pelvis to examine the prevalence of tumor types and clinical presentations in patients at or younger than 21 years of age.

## Methods

This is a retrospective cohort study of all patients who presented to the musculoskeletal program at the Children Hospital of Philadelphia and were diagnosed with pelvic sarcoma between January 2000 and June 2013. After approval from the Institutional Review Board, patients were identified from the Tumor Registry and the billing records. Patients were included if they had the diagnosis of pelvic bone or soft tissue sarcoma at age 21 years or younger. Patients had to have available data on tumor type and location even if presented for a second or third opinion consultation or were diagnosed and treated at a different institution. Patients were excluded if they had visceral pelvic sarcomas, testicular sarcoma, prostate sarcoma, or disseminated sarcoma.

Data on demographics, clinical presentation, tumor type and anatomic location, and metastatic status were collected from the medical records. Enneking classification was utilized to summarize tumor anatomic location [12]. Radiographs that were performed at presentation were reviewed to determine the tumor characteristics. If the radiographs were not available, we reviewed the radiographic report. Data was also collected on the geographic location as reported by the zip code.

Data was analyzed and presented using proportions and percentages for nominal variables and mean and standard deviation for continuous variables.

## Results

**Figure 1:**
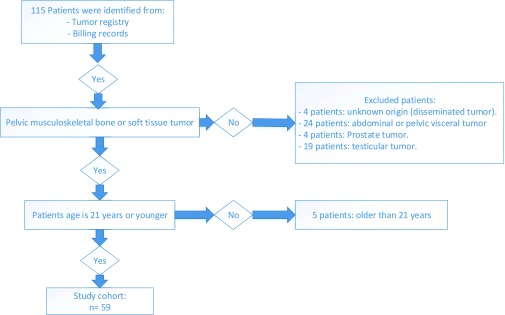
Schema illustrating the inclusion and exclusion criteria.

We identified 115 consecutive patients with pelvic bone or soft tissue malignant tumor, of these only 59 patients met the inclusion criteria and formed the study cohort (Figure 1). There were 37 males (62.7%) and 22 females (37.3%). Mean age at presentation was 11.3 ± 5.0 years (range 0.8–21 years). Twenty patients (33.9%) presented in the first decade and 39 patients (66.1%) presented in the second decade. Forty-three patients (72.9%) were White, 3 African American, 4 Hispanic, 2 Asian, and 2 were identified as other.

### Tumor types

**Figure 2:**
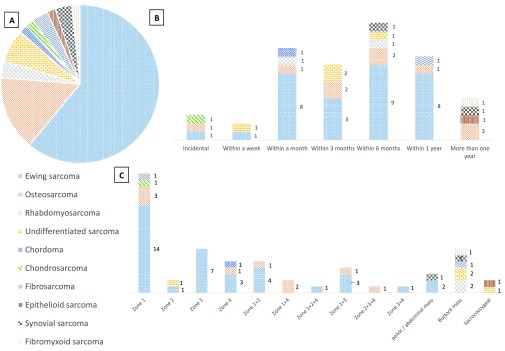
Patients' characteristics stratified by tumor type (A), time to presentation and tumor type (B), and anatomic location (Enneking zones) and tumor type (C).

Thirty-six patients had Ewing sarcoma (61%), 9 (15.2%) osteosarcoma, 4 (6.8%) undifferentiated sarcoma, 2 (3.4%) rhabdomyosarcoma, 2 (3.4%) synovial cell sarcoma, and 1 (1.7%) of each fibrosarcoma, dermatofibrosarcoma, fibromyxoid sarcoma, chondrosarcoma, chordoma, and epithelioid sarcoma (Figure 2). Most of the sarcomas were primarily bone tumors (49 cases) affecting one or more pelvic zones favoring the ilium (Figure 1). Ten patients had soft tissue sarcoma including 2 Ewing sarcoma, 2 rhabdomyosarcoma, 2 undifferentiated sarcoma, 2 synovial cell sarcoma, 1 dermatofibrosarcoma, and 1 fibromyxoid sarcoma.

### Clinical presentation

Pain was a presentation symptom in 41 patients (69%) (Table 1). Pain location was in the hip in 14 patients (34%), 7 leg, 3 groin, 2 back, 1 thigh, 1 buttock, 1 sacrum, and 1 pubis. Nine patients reported pain in more than one location, mostly the hip with other locations. Most of the bony tumors were painful (77%), while most of the soft tissue tumors were painless (70%).

**Table 1:** List of symptoms at presentation.

**Race **	
White	43
African American	3
Hispanic	4
Asian	2
Other	2
Not defined	5
**Symptoms at presentation **	
Pain	41
Mass	13
Buttock asymmetry	1
Thigh asymmetry	1
Leg or thigh weakness / numbness	5
Fatigue and other constitutional symptoms	9
Bowel and bladder changes	3
Limp	8
Incidental	3
**Period of symptoms before presentation **	
Within one week	1
Within one week to one month	11
Within 2 to 3 months	9
Within 4 to 6 months	14
Within 7 to 12 months	10
More than one year	4
**Tumor location **	
Ilium (Zone 1)	19
Acetabulum (Zone 2)	2
pubis / Ischium (Zone 3)	7
Sacral wing (Zone 4)	5
Zone 1 + 2	5
Zone 1 + 4	2
Zone 1 + 2 + 4	1
Zone 2 + 3	4
Zone 2 + 3 + 4	1
Zone 3 + 4	1
Sacorcoccygeal	2
Soft tissue (Buttock)	7
Soft tissue (pelvic mass)	3
**Metastases **	
Pulmonary	16
Bone marrow	4
To Bone	9

Thirteen patients (22%) presented with soft tissue mass, 5 were soft tissue tumors, and 8 were bone tumors. Mass location was 4 in the buttock, 2 hip, 2 pelvis, 1 thigh, 1 sacrum, 1 coccyx, and 1 groin. Three of them were associated with pain (2 Ewing sarcoma and 1 Chordoma).

Two patients presented with asymmetry in the buttocks or thighs (one undifferentiated sarcoma and one rhabdomyosarcoma). Eight patients presented with a limp. Five patients had neurologic symptoms including numbness, weakness, or radicular pain (3 Ewing sarcoma and 2 Osteosarcoma). Nine patients (7 Ewing sarcoma, 1 osteosarcoma, and 1 high-grade undifferentiated sarcoma) presented with constitutional symptoms including weight loss or fever and all had bony tumors.

Time between symptoms onset and presentation varied and was mostly within 4–12 months (Figure 2). Three patients presented incidentally (one had hip pain after he fell in a basketball game, one with history of bed-wetting at age 10 years, and one was known to be a 5 years survivor of femoral Ewing sarcoma and an iliac lesion was diagnosed during a regular clinic evaluation).

### Radiographic presentation

**Figure 3:**
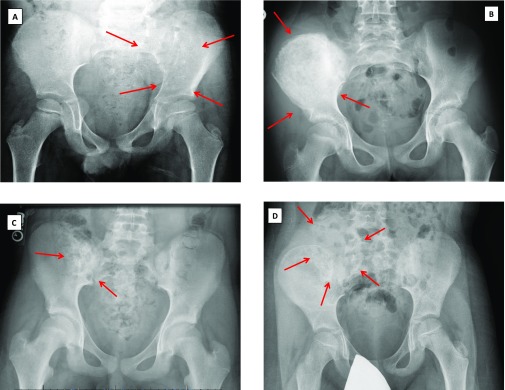
Radiographic presentation of pelvic sarcoma. Lytic lesion in 6.7-year-old male with Ewing sarcoma in the left ilium (A), calcific lesion in a 12 years old female with Ewing sarcoma in the right ilium (B), mixed lesion in a 14 years old male with Ewing sarcoma in the right ilium (C), and soft tissue mass without boney lesion in a 7 years old male with Ewing sarcoma (D).

Radiographs were available for only 30 patients (Table 2). The radiographic features of pelvic sarcoma varied between patients (Figure 3). Eleven (36.7%) presented with a lytic lesion, 10 Ewing sarcoma (3 with a soft tissue mass), and 1 chondrosarcoma. Four (13.3%) presented with a sclerotic lesion, 2 Ewing sarcoma, and 2 Osteosarcoma (both with a soft tissue mass). Six (20%) presented with a mixed sclerotic and lytic lesion (5 Ewing sarcoma and 1 osteosarcoma). A soft tissue mass without osseous lesion was noted in 6 patients (3 Ewing sarcoma and 1 of undifferentiated sarcoma, rhabdomyosarcoma, and synovial cell sarcoma). No osseous or soft tissue mass was reported in 2 patients (Ewing sarcoma and undifferentiated sarcoma). One patient with undifferentiated sarcoma had a pelvic radiograph without osseous or soft tissue sarcoma but showed osteopenia.

**Table 2:** Clinical and radiographic presentation stratified by sarcoma type.

Tumor type	*N*	Pain	Mass	Constitutional symptoms	Radiographic characteristics			
					
					Lytic lesion	Sclerotic lesion	Mixed lesion	Soft tissue only
Ewing Sarcoma	36	29	4	10	10	2	5	3
Osteosarcoma	9	9	0	2		2	1	
Rhabdomyosarcoma	2	0	1	0				1
Undifferentiated sarcoma	4	1	2	1				1
Chondrosarcoma	1	0	0	_	1			
Chordoma	1	1	1	0				
Fibrosarcoma	2	0	2	0				
Epithelioid sarcoma	1	0	1	0				
Synovial cell sarcoma	2	1	1	0				1
Fibromixoid sarcoma	1	0	1	0				

### Metastatic status

Twenty-one cases (35.6%) presented with metastasis [14 (39%) Ewing sarcoma, 6 (66.7%) osteosarcoma, and 1 (50%) synovial cell sarcoma], 16 cases to the lung, 9 to other bones (skull, ribs, femur, and vertebrae), and 4 to bone marrow (Table 3). Age at presentation in these cases was older than 9 years except one case of Ewing sarcoma (was 5 years), one osteosarcoma (was 4 years old), and one synovial cell sarcoma (was 4.6 years old). Time to presentation varied in these patients from one month to 9 months.

**Table 3:** Characteristics of metastatic pelvic sarcoma patients.

Age	Clinical presentation				Time to presentation	Location	Type	Metastases location		
	Pain	Mass	Constitutional symptoms	Limping				Lung	Bone marrow	Bone
M, 10.3	Yes	No	Yes		2 months	1+2+4	Ewing Sarcoma	Yes	No	No
M16.3	Yes	No	Yes		2 months	3	Ewing Sarcoma	Yes	No	No
M, 6.9	Yes	No	No		2 weeks	5	Ewing Sarcoma	Yes	No	No
F, 16.8	Yes	No	Yes		9 months	1	Ewing Sarcoma	Yes	No	Yes
M, 12.9	Yes	No	Yes		1 month	4	Ewing Sarcoma	No	No	Yes
F, 4.4	Yes	No	Yes		2 months	1+2	Ewing Sarcoma	Yes	Yes	Yes
M, 17.8	Yes	No	No		7 months	1+2	Ewing Sarcoma	Yes	No	No
M, 11.9	Yes	No	Yes		2 months	1	Ewing Sarcoma	No	Yes	No
M, 12.5	Yes	No	No		5 months	3	Ewing Sarcoma	Yes	No	Yes
F, 5.1	Yes	No	Yes		7 months	1	Ewing Sarcoma	No	No	Yes
F, 9.9	Yes	No	No		1 week	3	Ewing Sarcoma	Yes	No	No
M, 14.1	Yes	No	No		7 months	3	Ewing Sarcoma	Yes	No	No
M, 12.4	Yes	Yes	No		3 months	1+2	Ewing Sarcoma	Yes	No	No
F, 10.4	Yes	No	No		one month	3	Ewing Sarcoma	Yes	Yes	Yes
M, 9.9	Yes	No	No		> 6 months	2+3	Osteosarcoma	Yes	No	No
M, 9.7	Yes	No	No	Yes	5 months	1	Osteosarcoma	Yes	No	No
M, 18.5	Yes	No	No		incidental	1+4	Osteosarcoma	Yes	Yes	No
F, 12.3	Yes	No	Yes		6 months	2+3+4	Osteosarcoma	No	No	Yes
M, 3.9	Yes		No	Yes	1 month	1	Osteosarcoma	Yes	No	Yes
M, 15.2	Yes	No	No		2-3 months	1	Osteosarcoma	No	No	Yes
M, 4.6	No	Yes	No		3 years	Pelvic mass	Synovial cell sarcoma	Yes	No	No

Location was stratified based on Enneking classification.

### Geographic location

Based on zip-codes, most patients were living in the east coast and did not present a specific distribution in urban or rural areas.

### Patients' characteristics stratified by tumor types

#### Ewing sarcoma

Thirty-six patients had Ewing sarcoma (24 males and 12 females) with male to female ratio of 2:1. Mean age at presentation was 12.2 years, range from 9 months to 21 years. Fourteen patients presented with metastases (28.9%) (11 to the lung, 3 bone marrow, and 6 to other bones).

#### Osteosarcoma

There were 9 patients with osteosarcoma (8 males and 1 female), with mean age at presentation of 13 years, range from 3.9 to 18.5 years. In addition to pain, 1 patient developed bowel and bladder changes, 1 had thigh and leg numbness and weakness (both had constitutional symptoms), and 3 were limping at presentation. Six patients (66.7%) presented with metastases (4 to the lung, 3 to bones, and 1 to bone marrow).

#### Rhabdomyosarcoma

Two boys aged (1.3 and 2.3 years), one presented with a painless buttock mass and one with thighs asymmetry. None had metastases at presentation.

#### Undifferentiated sarcoma

Four patients (1 boy and 3 girls) presented at age between 9 months and 15 years. One of these patients presented with pain and constitutional symptoms, 2 presented with a mass (buttock and hip), and 1 presented with gluteal asymmetry. None of the patients presented with metastases.

#### Chordoma

One male patient presented at age of 15.9 years with painful sacral mass for about a month without metastases at the time of presentation.

#### Chondrosarcoma

One female patient presented at age of 10.9 years with the complaint of bed-wetting. The pelvic X-ray incidentally showed a lytic lesion in the ilium. The patient did not have metastases at the time of presentation.

#### Fibrosarcoma and dermatofibrosarcoma

Two females presented at 12.9 and 2.8 years of age with a painless buttock mass. The pathology in the older patient was dermatofibrosarcoma protuberans (DFSP) with areas of frank fibrosarcoma. The pathology in the younger was fibrosarcoma. Both patients had a localized disease with no metastases.

#### Epithelioid sarcoma

A 9.2 years old female presented with a painless coccygeal mass that persisted for more than 2 years. There was no metastasis at the time of presentation.

#### Synovial cell sarcoma

Two patients were diagnosed with synovial cell sarcoma. Eleven years old female presented with a 6 months history of hip and buttock pain. The other patient was a 4.6 years old male who presented with a 3-years history of inguinal painless slow growing mass associated with pulmonary metastasis.

#### Fibromyxoid sarcoma

An 11.9 years old male presented with a painless gluteal mass that had been there for 3 years. There was no metastasis at the time of diagnosis.

## Discussion

Children with pelvic sarcoma may present with symptoms that mimic other benign conditions. We have found that atraumatic pain and limping were common and lasted for few weeks to months prior to presentation. Hip pain and limping in children is a challenging condition and can be difficult to examine and determine the diagnosis. Age at presentation can be a diagnostic parameter for certain conditions. For example, a limping 5–9-year-old child is highly suspicious of Perthes disease while a limping adolescent can be related to Slipped Capital Femoral Epiphysis (SCFE) or diskitis [13].

In addition to pain, patients in our study presented with a mass, neurological symptoms, or bladder changes. We found that patients with Ewing sarcoma presented with or without a mass, while all patients with osteosarcoma presented with pain without a mass. We also found that most soft tissue sarcomas were painless and likely presented with a mass (5 of 10 patients, 50%) compared to bony sarcomas (8 of 49, 16%). Constitutional symptoms were not common at presentation as they were only seen in 9 patients (15%), mostly in patients with Ewing sarcoma (7 of 36 patients, 19%) which is consistent with previous reports. Frassica et al. reported presence of fever in 10 of 27 patients with pelvic Ewing sarcoma [14]. Bacci et al. reported 13% incidence of fever in Ewing sarcoma non-specific to pelvis [15].

Pelvic tumors are mostly deep and are close to other structures that cause vague and insidious presentation. The typical radiographic feature of malignant bone tumor is aggressive permeative lesion [16]. In our study, we found that the radiographic features of pelvic sarcoma varied between lytic, calcific (sclerotic), and mixed bony lesions. Some patients had normal pelvic radiographs. This was previously reported by Frassica et al. who reported normal pelvic radiographic finding in 5/27 patients (18.5%) [14].

Pelvic sarcoma patients may present late and therefore have higher rate of metastasis [17]. Our study showed that 21 (35.6%) patients presented with metastatic disease, almost all were older than 9 years of age, all were bony tumors (Ewing sarcoma and osteosarcoma) except one that was soft tissue tumor (synovial cell sarcoma). This is somewhat consistent with what was reported in the literature. Hoffman et al. reported 32% metastatic pelvic Ewing sarcoma at the time of diagnosis and most of them were in the second and third decades of age [18]. Ozaki et al. showed that 22.4% of pelvic osteosarcoma were metastatic at presentation and similarly most patients were older than 10 years [19]. Factors associated with poor survival in pelvic osteosarcoma are metastasis at presentation, tumor size (>10 cm) and location in the sacrum [20]. Similarly, in Ewing sarcoma, metastasis at presentation, central location, and poor response to chemotherapy lead to low survival [14,18]. Rhabdomyosarcoma and synovial cell sarcoma share similar prognostic factors in addition to histology type [3,21]. Bacci et al. examined patients with Ewing sarcoma family tumor that was not only located in the pelvis and reported increased risk of metastasis if patients presented with fever and if time to presentation was short [15]. We did not find this observation to be true in our study possibly because of small sample size and that our patient sample was limited to pelvic sarcoma.

We found in this study that Ewing sarcoma is the most prevalent bone sarcoma in the pelvis, which is similar to what has been reported [5,15]. We also reported other types of sarcoma in the pelvis. Fibromyxoid sarcoma is a low grade soft tissue tumor that mostly occurs in deep rather than subcutaneous tissues [22]. Fibrosarcoma is a rare soft tissue sarcoma (<1%) and has favorable outcome and is managed with surgical excision [23]. Wide resection is also adequate for dermatofibrosarcoma although it has local aggressive infiltration features [24]. Synovial cell sarcoma in children is mostly located in the lower extremity and has a 46% chance of being invasive [21]. Okcu et al. reported 21% rate of distant metastasis at diagnosis. Wide resection is a significant survival factor in synovial cell sarcoma [21].

The geographical distribution of the patients was mostly located in the east coast without a preference of rural areas which contradicts what has been reported previously [10]. Some reports indicated increased rate of children with Ewing sarcoma in farming families [25]. In our study, data on occupational exposure of the patients was limited.

This study has limitations due to the retrospective nature and relatively small sample size which limited the ability to examine low incidence tumors like fibrosarcoma, dermatofibrosarcoma, and chondrosarcoma. In addition, the nature of this study being retrospective limited our ability to retrieve all radiographs, specifically because some patients were referred to our department for a second or third opinion and radiographs were not available in the medical records. Lack of radiographs for all patients reduced the ability to perform powerful statistical analysis. We did not determine prognostic factors or any possible relation between survival and tumor type, location, or metastasis status. Regardless of these limitations, we were able to describe the clinical and radiographical presentation of pelvic sarcoma in children. Pain was the most common symptom in bony pelvic sarcomas. Soft tissue sarcomas were mostly painless at presentation. Pelvic osteosarcoma was painful without a soft tissue mass and tended to present with metastases. Constitutional symptoms were not common at presentation in pelvic sarcoma. Bone and soft tissue sarcomas are rare in children and adolescents, and should be considered as a differential diagnosis depending on the site of complaint.

## Conflict of interest

Dr. John Dormans reports occasional visiting professorship honorarium and or consulting fees. Drs. Muayad Kadhim, Nariman Abol Oyoun, and Richard Womer have no conflicts of interest to disclose.
